# Associations of the Fecal Microbial Proteome Composition and Proneness to Diet-induced Obesity*[Fn FN2]

**DOI:** 10.1074/mcp.RA119.001623

**Published:** 2019-07-01

**Authors:** Hao Q. Tran, Robert H. Mills, Nicole V. Peters, Mary K. Holder, Geert J. de Vries, Rob Knight, Benoit Chassaing, David J. Gonzalez, Andrew T. Gewirtz

**Affiliations:** ‡Center for Inflammation, Immunity and Infection, Institute for Biomedical Sciences, Georgia State University, Atlanta, GA; §Department of Pharmacology, University of California, San Diego, CA; ¶Skaggs School of Pharmacy and Pharmaceutical Sciences, University of California, San Diego, CA; ‖Department of Pediatrics, and Department of Computer Science and Engineering, University of California, San Diego, CA; **Center for Microbiome Innovation, University of California, San Diego, CA; ‡‡Neuroscience Institute, Georgia State University, Atlanta, GA; §§School of Psychology, Georgia Institute of Technology, Atlanta, GA 30332

**Keywords:** Mass Spectrometry, Omics, Obesity, Microbiome, Immunology*, Mouse models

## Abstract

Consumption of refined high-fat, low-fiber diets promotes development of obesity and its associated consequences. Although genetics play an important role in dictating susceptibility to such obesogenic diets, mice with nearly uniform genetics exhibit marked heterogeneity in their extent of obesity in response to such diets. This suggests non-genetic determinants play a role in diet-induced obesity. Hence, we sought to identify parameters that predict, and/or correlate with, development of obesity in response to an obesogenic diet. We assayed behavior, metabolic parameters, inflammatory markers/cytokines, microbiota composition, and the fecal metaproteome, in a cohort of mice (*n* = 50) prior to, and the 8 weeks following, administration of an obesogenic high-fat low-fiber diet. Neither behavioral testing nor quantitation of inflammatory markers broadly predicted severity of diet-induced obesity. Although, the small subset of mice that exhibited basal elevations in serum IL-6 (*n* = 5) were among the more obese mice in the cohort. While fecal microbiota composition changed markedly in response to the obesogenic diet, it lacked the ability to predict which mice were relative prone or resistant to obesity. In contrast, fecal metaproteome analysis revealed functional and taxonomic differences among the proteins associated with proneness to obesity. Targeted interrogation of microbiota composition data successfully validated the taxonomic differences seen in the metaproteome. Although future work will be needed to determine the breadth of applicability of these associations to other cohorts of animals and humans, this study nonetheless highlights the potential power of gut microbial proteins to predict and perhaps impact development of obesity.

## Abstract (precis)

Individuals exhibit marked heterogeneity in the degree of adiposity that results from consumption of an obesogenic diet. We hypothesized that markers of inflammation, fecal microbiome, and/or proteome composition might associate with heterogeneity in diet-induced obesity. We found the fecal proteome, which was comprised of microbial proteins that changed markedly upon exposure to the obesogenic diet associated with extent of obesity and associated dysglycemia. These results suggest the fecal microbial proteome plays a role in diet-induced obesity.

## Graphical Abstract

**Figure:**
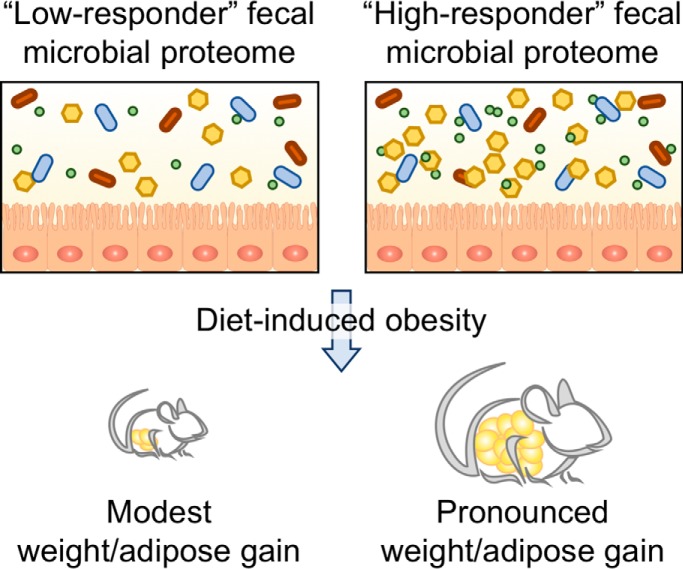

- Feeding mice a western-style obesogenic diet resulted in dramatic changes in fecal microbial proteome.
- Basal fecal microbial proteome, but not microbiome, composition was associated with extent of diet-induced obesity.
- A major feature of fecal microbial proteome of high-responder mice was enriched motility-related proteins.

Obesity is an emerging 21st century epidemic. Obesity, and the disease states it drives, including type 2 diabetes, cardiovascular disease, and liver disease threaten to overwhelm healthcare systems (1). Thus, obesity is a contemporary medical concern that poses a grave public health crisis in dire need of a solution. The increased incidence in obesity is thought to have been driven by broad societal changes that have resulted in reduced physical activity and increased availability of palatable low-cost energy-rich foods (2). Yet the extent to which individuals develop obesity in such an environment is highly heterogeneous. Variations in individual genetics contribute to, but are insufficient to fully explain, such heterogeneity. For example, studies characterizing weight-discordant monozygotic twins has shown that individuals with shared environmental, physical activity, and genetic factors display heterogeneity in adiposity (3). Similarly, rat-based studies show marked heterogeneity in weight gain and adiposity in response to obesogenic diets even when using highly inbred animals in a well-controlled environment (4, 5). Better understanding non-genetic factors that influence proneness to obesity might aid the identification of individuals at-risk for development of obesity and can yield modifiable factors to ameliorate this disease state.

Several factors that are at least partially independent of genetics are proposed to influence proneness to diet-induced obesity (DIO)^1^. One potential central nexus of such factors is inflammation, impacting metabolic signaling pathways including insulin and leptin (6), which have well-established roles in feeding behavior. Inflammation is also suggested to promote behavioral patterns such as anxiety-like and anti-social behaviors that can impact food consumption (7). Although numerous elements impact inflammation, one increasingly appreciated factor is the gut microbiota (8–12), which is the collective term for the large diverse community of microorganisms that inhabit the gastrointestinal tract. Indeed, in humans, gut microbiota composition is associated with obesity. One way microbiota composition influences metabolic signaling is via lipopolysaccharide (LPS), which activates pro-inflammatory signaling via Toll-like receptor 4 (TLR4) resulting in production of molecules including tumor necrosis factor alpha (TNF-α), and interleukin-6 (IL-6). These molecules interfere with leptin and insulin signaling, wherein LPS derived from gamma-proteobacteria is a particularly potent activator of TLR4 (13). Another host-microbiota interaction implicated in inflammation and obesity is the sensing of flagella through TLR5, which keeps motile bacteria in-check by a range of mechanisms including production of antimicrobial peptides and promoting production of anti-flagella immunoglobulins that help regulate the microbiota in the healthy gut (14). In addition to its impacts on inflammation, microbiota composition is also reported to influence energy harvest from ingested food (12, 15). Hence, considering its ability to impact inflammation, metabolism, and behavior, gut microbiota composition might provide a means of identifying host proneness to obesity when presented with an obesogenic diet.

Here, we sought to identify microbiota-based markers that might predict proneness to diet-induced obesity, specifically exposing mice to a western-style, low-fiber high fat diet (HFD). Both targeted and untargeted approaches were utilized including 16S rRNA gene amplicon sequencing for microbial community profiling and a Tandem Mass Tag (TMT) based multiplexed mass spectrometry (MS) approach for analysis of the fecal metaproteome. Additionally, we measured behavior, inflammatory markers, and metabolic parameters. Notably, we show that the fecal metaproteome appears to be a promising candidate for distinguishing mice with differential responses to obesogenic diets. Collectively, this study provides insight into potential mechanisms regarding the host-microbiota interactions mediating response to HFD exposure and highlights putative biomarkers for predicting DIO.

## EXPERIMENTAL PROCEDURES

### 

#### 

##### Mice and High-fat Diet Administration

Female, 3–5 week old C57BL/6 mice were purchased from Jackson Laboratory (Bar Harbor, ME) and maintained at Georgia State University, Atlanta, Georgia under institutionally approved protocol under approved protocols (IACUC # A14033), housed 5 mice per cage, were subjected to metabolic monitoring, including behavior analysis and sample collection over a 3-week period. During this time, the mice were fed standard grain-based chow (GBC), which is comprised of relatively unrefined ingredients. The cohort of mice was then switched to a diet composed of 60% kcal from fat (Research Diet, D12492) for 8 weeks. Mice were then euthanized, and colon length, colon weight, spleen weight and adipose weight were measured. Serum, feces, and organs were collected for downstream analysis.

##### Fecal Metaproteome Data Acquisition

Fecal samples were measured out to ∼0.2 g and suspended in 10 ml of ice-cold, sterilized TBS. A 20 μm vacuum, steriflip (Milipore, Burlington, MA) filter was used to remove particulate from the samples. Cells were pelleted through centrifugation at 4000 rpm for 10 min. Next, cells were lysed in 2 ml of buffer containing 75 mm NaCl (Sigma, St. Louis, MO), 3% sodium dodecyl sulfate (SDS, Fisher, Fair Lawn, NJ), 1 mm NaF (Sigma), 1 mm beta-glycerophosphate (Sigma), 1 mm sodium orhtovanadate (Sigma), 10 mm sodium pyrophosphate (Sigma), 1 mm phenylmethylsulfonyl fluoride (PMSF, Sigma), and 1X Complete Mini EDTA free protease inhibitors (Roche, Indianapolis, IN) in 50 mm HEPES (Sigma), pH 8.5 (16). An equal volume of 8 m Urea in 50 mm HEPES, pH 8.5 was added to each sample. Cell lysis was achieved through two 10 s intervals of probe sonication at 25% amplitude. Proteins were then reduced with dithiothreitol (DTT, Sigma), alkylated through iodoacetamide (Sigma), and quenched as previously described (17). Proteins were then precipitated via chloroform-methanol precipitation and protein pellets were dried (18). Protein pellets were re-suspended in 1 m urea in 50 mm HEPES, pH 8.5 and digested overnight at room temperature with LysC (Wako, Osaka, Japan) (19). A second, 6-hour digestion using trypsin at 37 °C was performed, and the reaction was stopped through addition of 10% trifluoroacetic acid (TFA, Pierce, Waltham, MA). Samples were then desalted through C18 Sep-Paks (Waters, Milford, MA) and eluted with a 40 and 80% Acetonitrile solution containing 0.5% acetic acid (20). Concentration of desalted peptides was determined with a BCA assay (Thermo Scientific, Rockford, IL). Fifty microgram aliquots of each sample were dried in a speed-vac, additional bridge channels consisting of 25 μg from each sample were created and 50 μg aliquots of this solution were used in duplicate per TMT 10-plex (Thermo Scientific) as previously described (21). These bridge channels were used to control for labeling efficiency, inter-run variation, mixing errors and the heterogeneity present in each sample (22). Each sample or bridge channel was resuspended in 30% dry acetonitrile in 200 mm HEPES, pH 8.5 for TMT labeling with 7 μl of the appropriate TMT reagent (23). Reagents 126 and 131 (Thermo Scientific) were used to bridge between mass spec runs. Remaining reagents were used to label samples in random order. Labeling was carried out for 1 h at room temperature and quenched by adding 8 μl of 5% hydroxylamine (Sigma). Labeled samples were acidified by adding 50 μl of 1% TFA. After TMT labeling, each 10-plex experiment was combined and desalted through C18 Sep-Paks and dried in a speed-vac. Each 10-plex experiment was fractionated using a High pH Reversed-Phase Peptide Fractionation Kit (Pierce) per manufacturer instructions. All LC-MS^2^/MS^3^ experiments were carried out on an Orbitrap Fusion (Thermo Fisher Scientific) with an in-line Easy-nLC 1000 (Thermo Fisher Scientific) and chilled autosampler. Separation and acquisition settings were as previously defined (24).

##### Metaproteome Data Processing

Data was processed using Proteome Discoverer 2.1 (Thermo Fisher Scientific). MS^2^ data was searched against a catalogue of mouse gut genes (25) (accessed 02/12/2017) containing 2,569,907 entries along side the Uniprot mouse proteome (www.uniprot.org, access date 11/14/2016) which contained 53,374 entries. The Sequest searching algorithm (26) was used to align spectra to database peptides. A precursor mass tolerance of 50 ppm (27, 28) was specified and 0.6 Da tolerance for MS^2^ fragments. Included in the search parameters was static modification of TMT 10-plex tags on lysine and peptide n-termini (+229.162932 Da), carbamidomethylation of cysteines (+57.02146 Da), and variable oxidation of methionine (+15.99492 Da). The search parameters included trypsin as the enzyme used to generate peptides with a maximum of 2 missed cleavages permitted. A two-step database search method was utilized (29) wherein proteins identified in either the forward or reverse database were included in a second search containing 14,368 entries from the original mouse gut gene catalogue, and annotations derived from this database were used for downstream analysis of microbial proteins (25). A peptide and protein false discovery rate of 1% was enforced using a reverse database search strategy (30–32).

TMT reporter ion intensities were extracted from MS^3^ spectra for quantitative analysis and signal-to-noise ratios were used for quantitation. Additional stringent filtering was used removing any moderate confidence peptide spectral matches (PSMs), or ambiguous PSM assignments. Additionally, any peptides with a spectral interference above 25% were removed, as well as any peptides with an average signal to noise ratio less than 10. Normalization occurred as previously described (24). Briefly, relative abundances are normalized first to the pooled standards for each protein and then to the median signal across the pooled standard. An average of these normalizations was used for the next step. To account for slight differences in amounts of protein labeled, these values were then normalized to the median of the entire dataset and reported as final normalized summed signal-to-noise ratios per protein per sample.

##### Behavioral Analysis

Three weeks after arrival in the animal facilities, behavior in the open field and in the home cage was assessed in a counter-balanced fashion over the course of 2 days. Behavioral testing occurred within the last 4 h of the light and quiescent phase and was conducted under illumination of overhead white lighting (between 300 and 400 lux). Open field arenas were cleaned with 70% ethanol between trials, and home cage bedding was changed after each trial. Behavioral tests were videotape using a Sony camcorder for later analysis by The Observer version XT11 (Noldus Information Technology Inc., Wageningen, The Netherlands). An experiment blinded as to the treatment conditions scored behavioral tests in the Observer.

##### Open Field Test

Locomotor behavior was assessed in a 43.2 X 43.2 X 30.5 cm (WxLxH) Plexiglas arena (Med Associates, Inc., St. Albans, VT) containing 2 arrays of infrared transmitters strips (16 beams each) located on the bottom of the arena (in the X and Y plane). The center zone of the arena was defined as square containing the center 8 beams (*e.g.* beams 4–12) in the X and Y plane. Each mouse was placed into the arena with its nose facing the wall and allowed to freely investigate for 10 min. The total distance traveled, the total time spent in the center of the arena, and circling behaviors, which are defined as movements below a preset ambulatory threshold, were calculated by Activity Monitor (Med Associates, Inc.) on a computer connected to the open field arenas.

##### Home Cage Behavior

Mice were placed into a clean housing cage containing 2 cm deep Alpha-dri bedding (Shepherd Specialty Paper, Fibercore, Cleveland, OH) and video recorded for 10 min. An experimenter blinded as to condition scored the occurrence and duration of (1) the time spent walking, as defined by locomotion along the bottom of the enclosure, around the arena, (2) grooming, as defined by stroking or scratching the face of body, (3) digging, as defined as using the fore- or hind paws to displace the bedding, and (4) the rears, as defined by standing on the hind legs with either the forepaws unsupported or when the forepaws were supported by the walls of the enclosure, were quantified using the Observer.

##### Fasting Blood Glucose Measurement and Body Composition Measurement

For fasting blood glucose tolerance test, mice were fasted for 5 h, and baseline blood glucose were measured by using a Nova Max plus Glucose meter and expressed in mg/dL. Measurement of percent fat mass and lean mass was performed via MRI (Bruker MiniSpec) at day 0, prior to diet treatment, and day 28 and 56, after diet treatment.

##### Fecal Sample Preparation for Immunoglobulin Quantification

Fecal sample preparation of enzyme-linked immunosorbent assay (ELISA) has been previously described (33). One hundred milligrams of fecal pellets were homogenized in 3 ml of collection media consisting of 0.05 mg soybean trypsin inhibitor per ml of a 3:1 mixture of 1× PBS and 0.1 m EDTA, pH 7.4. Following centrifugation at 1800 rpm for 10 min, the supernatant was centrifuged again at 14,000 rpm for 15 min at 4 °C, and final supernatant was collected and stored with 20% glycerol and 2 mm phenylmethylsulfonyl fluoride (Sigma, P-7626) at −20 °C until analysis.

##### Fecal and Serum Anti-flagellin IgA/IgG

Quantification of anti-flagellin- specific IgA and IgG has been previously described (34–36). Briefly, 96-well microtiter plates (Costar, Corning, NY) were coated with 100 ng/well of laboratory-made flagellin in 9.6 pH bicarbonate buffer overnight at 4 °C. Serum samples from mice were then applied either pure or at a 1:100 dilution for 1 h at 37 °C. After incubation and washing, the wells were incubated with either horseradish peroxidase-linked anti-mouse IgG (GE Healthcare Life Sciences, Pittsburgh, Pennsylvania) or horseradish peroxidase-linked anti-IgA (Southern Biotech, Birmingham, Alabama). Quantification of immunoglobulin was then developed by the addition of 3,3′,5,5′-Tetramethylbenzidine and the optical density was calculated by the difference between readings at 450 nm and 540 nm.

##### Fecal Lcn-2 Quantification

As previously described (37), frozen fecal samples were reconstituted in PBS containing 0.1% Tween 20 at 100 mg/ml and vortexed for 20 min. The homogenate was then centrifuged at 12,000 rpm for 10 min at 4 °C. Clear supernatants were collected and stored at −20 °C until analysis. Lcn-2 levels were measured in the supernatants using Duoset murine Lcn-2 ELISA kit (R&D Systems, Minneapolis, MN).

##### Myeloperoxidase Quantification

Tissue samples were homogenized in 100 mg/ml of 0.5% hexadecyltrimethylammonium bromide (Sigma, St. Louis, MO) in 50 mm PBS, pH 6.0, as previously described (37). Following 3 cycles of freeze-thaw at −80 °C and 37 °C, samples were sonicated and centrifuged at 14,000 rpm for 15 min at 4 °C. Supernatants were stored at −20 °C until analysis. Myeloperoxidase (MPO) was assayed in the supernatant by adding 1 mg/ml of dianisidine dihydrochloride (Sigma, St. Louis, MO) and 5 × 10^−4^% H_2_O_2_ and the change in optical density measured at 450 nm.

##### Serum CXCL1 and IL-6 Quantification

Serum chemokine (C-X-C motif) ligand 1 (CXCL1) and Interleukin-6 (IL-6) concentrations were determined using Duoset cytokine ELISA kits (R&D Systems, Minneapolis, MN) according to manufacturer's instructions (37).

##### Fecal Flagellin and Lipopolysaccharide Load Quantification

We quantified flagellin and lipopolysaccharide (LPS) as previously described using human embryonic kidney (HEK)-Blue-mTLR5 and HEK-BluemTLR4 cells, respectively (Invivogen, San Diego, CA) (37, 39). We resuspended fecal material in PBS to a final concentration of 100 mg/ml and homogenized for 10 s using a Mini-Beadbeater-24 without the addition of beads to avoid bacteria disruption. We then centrifuged the samples at 8000 × *g* for 2 min, serially diluted the resulting supernatant, and applied to mammalian cells. Purified *E. coli* flagellin and LPS (Sigma, St Louis, MO) were used for standard curve determination using HEK-Blue-mTLR5 and HEK-Blue-mTLR4 cells, respectively. After 24 h of stimulation, we applied cell culture supernatant to QUANTI-Blue medium (Invivogen, San Diego, CA) and measured alkaline phosphatase activity at 620 nm after 30 min.

##### Microbiota Analysis by 16S rRNA Gene Sequencing Using Illumina MiSeq Technology

16S rRNA gene amplification and sequencing were performed using the Illumina MiSeq technology following the protocol of Earth Microbiome Project with their modifications to the MOBIO PowerSoil DNA Isolation Kit procedure for extracting DNA (www.earthmicrobiome.org/emp-standard-protocols). Bulk DNA were extracted from frozen extruded feces using a PowerSoil-htp kit from MoBio Laboratories (Carlsbad, CA) with mechanical disruption (bead-beating). The 16S rRNA genes, region V4, were PCR amplified from each sample using a composite forward primer and a reverse primer containing a unique 12-base barcode, designed using the Golay error-correcting scheme, which was used to tag PCR products from respective samples (40). We used the forward primer 515F 5′-*AATGATACGGCGACCACCGAGATCTACAC***TATGGTAATT*GT***GTGCCAGCMGCCGCGGT AA-3′: the italicized sequence is the 5′ Illumina adapter B, the bold sequence is the primer pad, the italicized and bold sequence is the primer linker and the underlined sequence is the conserved bacterial primer 515F. The reverse primer 806R used was 5′-*CAAGCAGAAGACGGCATACGAGAT* XXXXXXXXXXXX **AGTCAGTCAG** ***CC**GGACTACHVGGGTWTCTAAT*-3′: the italicized sequence is the 3′ reverse complement sequence of Illumina adapter, the 12 X sequence is the golay barcode, the bold sequence is the primer pad, the italicized and bold sequence is the primer linker and the underlined sequence is the conserved bacterial primer 806R. PCR reactions consisted of Hot Master PCR mix (Five Prime), 0.2 μm of each primer, 10–100 ng template, and reaction conditions were 3 min at 95 °C, followed by 30 cycles of 45 s at 95 °C, 60 s at 50 °C and 90 s at 72 °C on a Bio-Rad thermocycler. Four independent PCRs were performed for each sample, combined, purified with Ampure magnetic purification beads (Agencourt), and products were visualized by gel electrophoresis. Products were then quantified (BIOTEK Fluorescence Spectrophotometer) using Quant-iT PicoGreen dsDNA assay. A master DNA pool was generated from the purified products in equimolar ratios. The pooled products were quantified using Quant-iT PicoGreen dsDNA assay and then sequenced using an Illumina MiSeq sequencer (paired-end reads, 2 × 250 bp) at Cornell University, Ithaca.

##### 16S rRNA Gene Sequence Analysis

Forward and reverse Illumina reads were joined using the fastq-join method (41, 42), sequences were demultiplexed, quality filtered using Quantitative Insights Into Microbial Ecology (QIIME, version 1.8.0) software package (43). QIIME default parameters were used for quality filtering (reads truncated at first low-quality base and excluded if: (1) there were more than three consecutive low quality base calls (2), less than 75% of read length was consecutive high quality base calls (3), at least one uncalled base was present (4), more than 1.5 errors were present in the bar code (5), any Phred qualities were below 20, or (6) the length was less than 75 bases). Sequences were clustered to operational taxonomic units (OTUs) using UCLUST algorithm (44) with a 97% threshold of pairwise identity (without the creation of new clusters with sequences that do not match the reference sequences), and taxonomically classified using the Greengenes reference database 13_8 (45). A single representative sequence for each OTU was aligned and a phylogenetic tree was built using FastTree (46). The phylogenetic tree was used for computing the unweighted UniFrac distances between samples (47, 48), rarefaction was performed and used to compare abundances of OTUs across samples. Principal coordinates analysis (PCoA) plots were used to assess the variation between experimental group (beta diversity). Alpha diversity curves were determined for all samples using the determination of the number of observed species. LEfSE (LDA Effect Size) was used to investigate bacterial members that drive differences between groups (49). Unprocessed sequencing data are deposited in the European Nucleotide Archive under accession number PRJEB33328.

### Experimental Design and Statistical Rationale

#### 

##### Study Design

The overall study included 50 mice which were followed longitudinally for monitoring of weight gain and other measures. The sample size was determined for statistical power based on previous publications (4, 5) and experience. The metaproteome analysis included four mice with the highest weight gain and the lowest weight gain. These samples sizes were determined to be enough based on previous reports of strong differences in related animal models with similar sample sizes (50).

##### Metaproteome Analysis

Metaproteome analysis was performed using python (version 3.5) and records are available online (https://github.com/rhmills/High-Fat_Diet_Metaproteomics_analysis). Extra files associated with the analysis within the notebooks are deposited as supplementary files in the MassIVE (https://massive.ucsd.edu) repository for this study (Study ID: MSV000083891). All analysis was performed on the proteins identified in both TMT 10-plex experiments. Qiime2, version 2019.1 (https://qiime2.org/), was used for principle coordinates analysis through the command “qiime diversity core-metrics” as well as for determining significance of beta-diversity clustering through the command “qiime diversity beta-group-significance.” K-means clustering was performed through Morpheus (https://software.broadinstitute.org/morpheus). Enriched and depleted proteins were determined by π–score, which accounts for both fold change and *p* value (51). A statistical cutoff for highly ranked associations was set to π > 1 (α ∼ 0.05), which provided an adequate number of proteins for functional and taxonomic assessment (52) while maintaining a moderate stringency. Volcano plots were visualized using GraphPad Prism (version 7.0b). Mouse protein gene functional enrichment analysis was performed using DAVID (53), with all mouse proteins identified as a background list. The python package, Seaborn (version 0.9.0) was used for boxplots, swarmplots, and catplots. Statistical analysis between groups within the boxplots was performed using ANOVA with Dunnett corrected *p* values through GraphPad Prism (version 7.0b).

##### Statistical Analysis

Significance was determined using unpaired two-tailed *t* test or linear regression analysis (GraphPad Prism software, version 6.01). Differences were noted as significant *p ≤ 0.05 for *t* test or linear regression analysis. For clustering analyzing on principal coordinate plots, categories were compared, and statistical significance of clustering was determined *via* Permanova (54).

## RESULTS

### 

#### 

##### Stratification and Characterization of Mice Prone, or Resistant, to HFD-induced Metabolic Syndrome

**Fig. 1.**
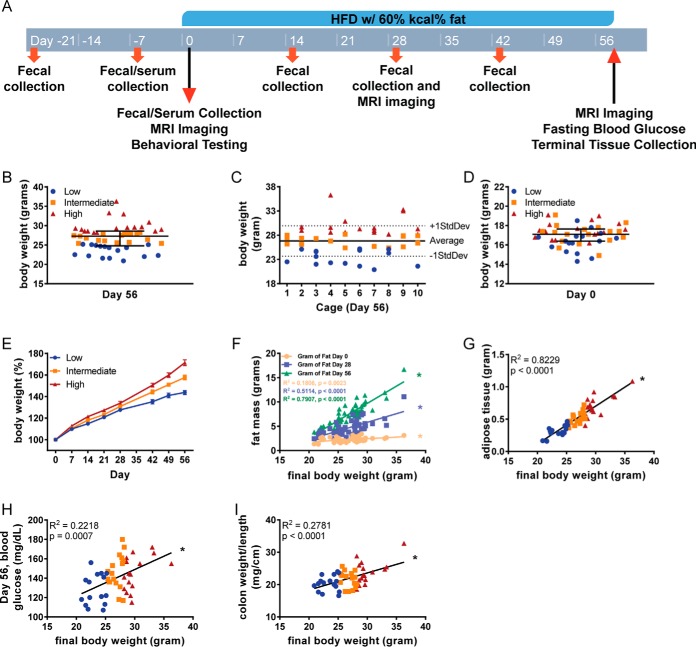
**Stratification and characterization of mice prone, or resistant, to HFD-induced metabolic syndrome.** *A*, 3–5-week old, female C57BL/6 mice were purchased from The Jackson Laboratory and housed for 3 weeks before high-fat diet administration in order to favor microbiota stabilization. Subsequently, animals were treated with high-fat diet (60% kcal from fat) for 8 weeks. Serum collection occurred on days −7, 0, and 56. Body weight measurements occurred prior to every flagellin administration. Fecal collection occurred on days −21 and −7, then every other week starting on day 0. *B*, Mice were identified as low, intermediate, or high responders based on if their final body weight fell within the first, second, or third tertile, respectively. *C*, Final body weights of mice by cage. *D*, Initial weights of mice. *E*, Body weights were measured weekly and expressed as relative values, day 0 (pre high-fat diet treatment) being defined as 100%. Final body weights were correlated to (*F*) fat mass by MRI, *G*, epididymal adipose weight, *H*, day 56 fasting blood glucose, and *I*, colon weight/length ratio. Data are the means ± S.E. (*n* = 50). Significance was determined using linear regression analysis (*p ≤ 0.05).

The primary goal of this study was to elucidate factors that predict and possibly govern, susceptibility to developing obesity in response to administration of an obesogenic diet. Hence, we designed a prospective study wherein 50, 3-week-old female C57BL/6 mice, housed 5 mice per cage, were subjected to metabolic monitoring, including behavior analysis and sample collection over a 3-week period. During this time, the mice were fed standard grain-based chow (GBC), which is comprised of relatively unrefined ingredients. The cohort of mice was then switched to a diet compositionally low in fiber (5%) and high in fat (35% by mass, 60% by calories), herein referred to as an obesogenic diet or high-fat diet (HFD) for an 8-week period. Prior to, during and after administration of the high-fat diet, sample collection and monitoring was performed as outlined in Fig. 1*A*. In accord with our previous rodent-based studies (5), the extent of obesity following the obesogenic diet was quite heterogeneous with many mice weighing between 20–25 grams, which is the approximate weight of age-matched GBC-fed mice of this strain/gender. In contrast, some mice appeared to dramatically gain weight over the course of the experiment with final weights over 30 grams. Therefore, based on their final body weight, mice were stratified into tertiles as being prone, intermediate, or relatively resistant to being obese following exposure to an obesogenic diet (Fig. 1*B*). First, we examined if mice prone or resistant to DIO clustered within cages but did not observe a distribution pattern to support this possibility (Fig. 1*C*). Nor were these groupings significantly related to the initial weight of the mice (Fig. 1*D*). The total weight gain over the period of exposure to the obesogenic diet for resistant mice was about 40%. This observation is approximately the expected age-related weight gain of GBC-fed mice during this period, whereas prone mice increased in weight by about 70% during this period (Fig. 1*E*). Fat mass, as determined by magnetic resonance imaging (MRI), prior to, during, or at the end of exposure to HFD, was highly correlated with body weight within the cohort (Fig. 1*F*). Accordingly, post-euthanasia weight of the periovarian fat pad, which has classically been used to assess adiposity in mice correlated closely (r^2^ = 0.8229) with final body weights confirming our stratifications reflected degree of adiposity (Fig. 1*G*). Final body weights were also correlated with fasting glucose concentration (r^2^ = 0.2218), suggesting mice that were prone to diet-induced obesity were also prone to its downstream consequences (Fig. 1*H*). Last, considering the appreciation that low-grade intestinal inflammation can promote adiposity and its consequences, we measured weight/length ratio of the colon (55, 56). This measurement was also correlated with final body weight (r^2^ = 0.2781; Fig. 1*I*), supporting the notion that the obese mice were in a state of low-grade gut inflammation.

##### Associations of Inflammatory Markers/Mediators and Proneness to Obesity

**Fig. 2.**
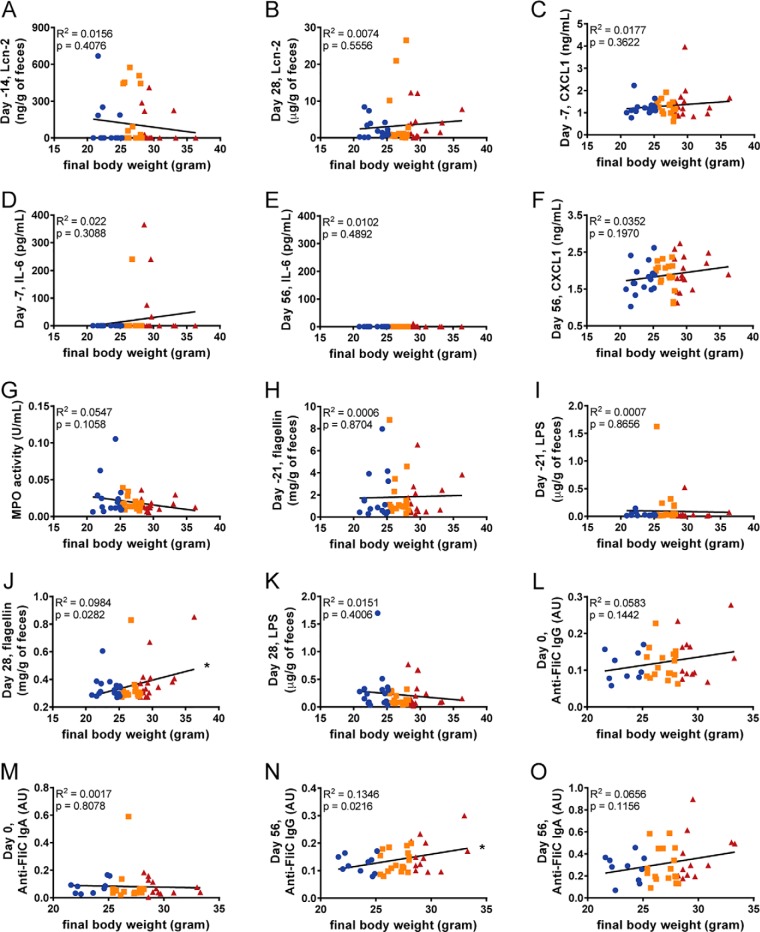
**Associations of inflammatory markers/mediators and proneness to obesity.** Final body weights correlated to fecal lipocalin-2 at (*A*) day −14 and (*B*) 28, analyzed using ELISA kits. Additionally, final body weights were correlated to serum cytokines CXCL1 and IL-6 at (*C–D*) day −7 and (*E–F*) day 56, analyzed using ELISA kits. Final body weights were also correlated to (*G*) colonic myeloperoxidase levels, as well as, fecal flagellin and lipopolysaccharide at (*H–I*) day −21 and (*J–K*) day 28 using HEK 293 cells expressing mTLR5 or mTLR4 measuring bioactive flagellin and lipopolysaccharide, respectively. Serum anti-flagellin IgG and IgA were also quantified using ELISA techniques at days 0 (*L–M*) and 56 (*N–O*). (*n* = 50). Significance was determined using linear regression analysis (*p ≤ 0.005).

Low-grade inflammation is reported to associate with, and promote obesity (56, 57). Accordingly, we investigated levels of pro-inflammatory mediators to determine if they might mark mice that would be prone to becoming obese following exposure to an obesogenic diet. Hence, we measured levels of fecal lipocalin-2 (Lcn-2), which is a broadly dynamic marker of gut inflammation (37). Levels of fecal Lcn-2 did not correlate with final body weights when measured 14 days prior (r^2^ = 0.0156) to exposure or 4 weeks after the initiation of the diet (r^2^ = 0.0074; Fig. 2*A*, 2*B*). Additionally, the levels of serum pro-inflammatory cytokines CXCL1 and IL-6 when measured 7 days prior to administration of the obesogenic diet were also not correlated to final body weight (r^2^ = 0.0177, 0.022 respectively, Fig. 2*C*, 2*D*). However, 4 of the 5 mice that displayed detectable serum IL-6 at this time point were in the top tertile of obesity following the diet suggesting the subset of mice displaying this parameter might be more prone to DIO. To further investigate this subset of mice, we tested for differences within various parameters associated with diet-induced obesity between the subsets of mice with or without detectable IL-6. At day −7, several of these parameters were consistent with the possibility that detection of IL-6 can discriminate high or low responders, but ultimately, none reached statistical significance (supplemental Fig. S1). Nevertheless, such elevations in IL-6 were not maintained when assayed after 8-weeks of diet (Fig. 2*E*). Other findings supporting the notion that obesity is associated with low-grade inflammation included a modest correlation after 8-weeks of diet between body weights and CXCL1, which is a chemokine expressed by many cell types and often used as a general serum marker of low-grade inflammation (r^2^ = 0.0352, Fig. 2*F*). In contrast, there was no correlation between final body weights and levels of intestinal myeloperoxidase (MPO), which is a widely used marker of classic inflammation in the intestine (58) (Fig. 2*G*).

Gut bacterial components, flagellin and lipopolysaccharide (LPS), are well known for their inflammatory properties (59, 60). Fecal levels of each were measured 3 weeks prior to administration of the obesogenic diet with neither correlating with final body weight (Fig. 2*H*, 2*I*). However, flagellin, but not LPS, were modestly correlated with final body weight when measured 4 weeks following initiation of the obesogenic diet (r^2^ = 0.0984, 0.0151 respectively, Fig. 2*J*, 2*K*). Moreover, there was a correlation in levels of anti-flagellin antibodies at the time of diet administration (for IgG but not IgA) and 8 weeks following exposure to obesogenic diet (Fig. 2*L*–2*O*). Levels of anti-flagellin antibodies likely reflect exposure of the immune system to this molecule, which can be influenced by both levels of flagellin in the gut, bacterial-epithelial distance, and intestinal permeability (35, 61). Together, these studies did not reveal a reliable predictive marker of proneness to diet-induced obesity but suggest exposure to bacterial products, such as flagellin, might have some predictive power.

##### Quantitative Measures of Behavior Did Not Predict Obesity Proneness

The gut-brain axis is increasingly appreciated to play a role in the pathogenesis of many neurological and metabolic diseases (62). Hence, we investigated the extent to which certain behavioral parameters are able to predict proneness to weight gain. Compulsive behavior and activity level were measured in a home cage behavior test, and time spent digging (supplemental Fig. S2*A*), time spent grooming (supplemental Fig. S2*B*), and total distance traveled (supplemental Fig. S2*C*) were quantified. Additionally, anxiety-like behavior was assessed using the open field test, represented by time spent in the center zone (supplemental Fig. S2*D*) and distance traveled in the center (supplemental Fig. S2*E*) of the open field arena. Ultimately, none of these measures had a significant ability to predict extent of obesity in response to the obesogenic diet.

##### Impact of DIO on Fecal Metaproteome

We next turned to a contemporary metaproteomics approach to study the fecal protein composition of our cohort of mice. Although administration of an obesogenic diet is well known to rapidly alter gut microbiota species composition (63), whether it might also impact the fecal metaproteome, let alone whether the fecal metaproteome might predict responsiveness to such a diet, has not been described. Although metaproteomic analysis presents the challenges of discriminating host and bacterial proteins from potentially millions of proteins, the field is an area of rapid growth currently developing standard methodology (64).

To this end, we applied our recently developed TMT-based metaproteomic methods (52), in combination with a two-step database search method (29) on feces from mice that developed the highest and lowest degree of obesity (*n* = 4 mice per condition). Our analysis included specimens collected before (day 0) and after 56 days of exposure to the obesogenic diet. The final data included quantitation of 13,975 proteins of which 1,108 were derived from mice (supplemental Table S1).

**Fig. 3.**
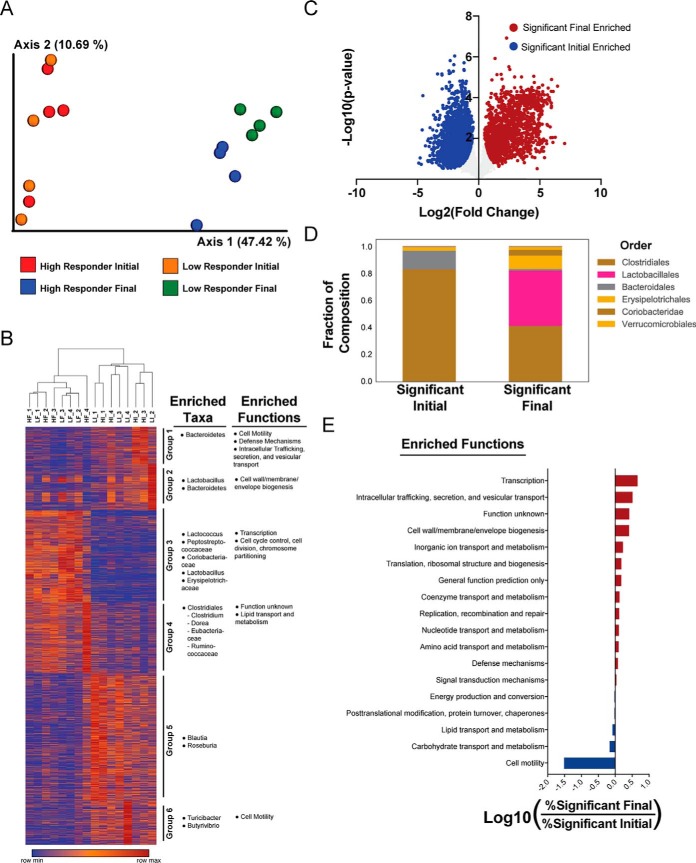
**Impact of DIO on fecal metaproteome.** *A*, Principal coordinates analysis (PCoA) of metaproteome data using the Bray-Curtis distance metric *B*, Protein relative abundance heatmap. Samples are clustered by 1-Pearson correlation and proteins are grouped using KMeans clustering. Relative abundances per protein are colored on a spectrum with red as row maxima and blue as row minima. Functional and taxonomic bias within each KMeans cluster is displayed on the right. *C*, Volcano plot of metaproteome response to HFD. Fold change and *t* test significance of each protein are plotted. Overall significance was set at π-score > 1. *D*, Taxonomic composition of significant proteins. *E*, Functional bias in significant proteins. Compositions of eggNOG annotations between proteins enriched in the final and initial time points were compared and the log ratio of high abundance categories (>10 proteins) is shown. Sample names in (*A–B*) are annotated H for High Responder, L for Low Responder, then I for Initial time point, F for Final time point, with 1, 2, 3, or 4 for replicate number.

For a broad scale perspective of the data, an unsupervised Principle Coordinates analysis of the metaproteome data using the Bray-Curtis distance metric exhibited clear separation of samples before and after diet administration, reflecting a dramatic impact of the obesogenic diet on the overall fecal metaproteome (Fig. 3*A*). This analysis also exhibited clustering at the 56-day time point discriminating high and low response to diet (Permanova pseudo-F = 1.99, *p* = 0.058). Using K-means clustering, we identified 6 protein clusters, some of which were associated with increased representation of taxa and functions (Fig. 3*B*). These groupings include Group 4, which appeared to show an increased presence of Clostridiales and lipid transport and metabolism proteins in high responder mice after exposure to HFD (Fig. 3*B*). These groupings provide putative taxonomic associations to the functional differences observed before and after administration of HFD.

Comparing all samples before and after HFD exposure made evident that there were widespread changes in the fecal metaproteome. By using a statistical ranking method accounting for both fold change and *t* test *p* values, π-score (51), we observed that 58% (3670/6311) of proteins displayed a high level of association to diet exposure (π > 1, Fig. 3*C*). The proteins associated with the dietary intervention contained large differences in their taxonomic and functional annotations. Taxonomic differences included a larger portion of proteins from Clostridiales and Bacteroidales before HFD exposure whereas a large portion (∼40%) of proteins enriched after 8-weeks exposure were derived from Lactobacillales (Fig. 3*D*).

Functional categorization of the proteins associated to the dietary intervention was performed through the Evolutionary Genealogy of Genes: Non-supervised Orthologous Groups (eggNOG) database. These studies revealed a very strong association between proteins related to motility and HFD exposure as expression levels of 141 proteins were reduced following exposure to HFD with only 3 proteins increased after HFD, resulting in a 32-fold difference (Fig. 3*E*). In accord, we note that, on average, levels of fecal flagellin decreased by about 5-fold when measured 21 days preceding or 4 weeks following administration of the obesogenic diet (Fig. 2*H*, 2*J*). Further, when subsetting all 680 flagellin proteins from the metaproteome dataset, we observed statistically significant decreases in abundance for both high and low responding mice (*p* < 0.0001; supplemental Fig. S3*A*). Functional assessment of the proteins enriched after HFD exposure resulted in weaker associations, the strongest of which was a 1.5-fold increased representation of Transcription proteins (Fig. 3*E*).

##### Functional and Taxonomic Characterization of Fecal Metaproteome in Low- and High-responder Mice Fed the Obesogenic Diet

**Fig. 4.**
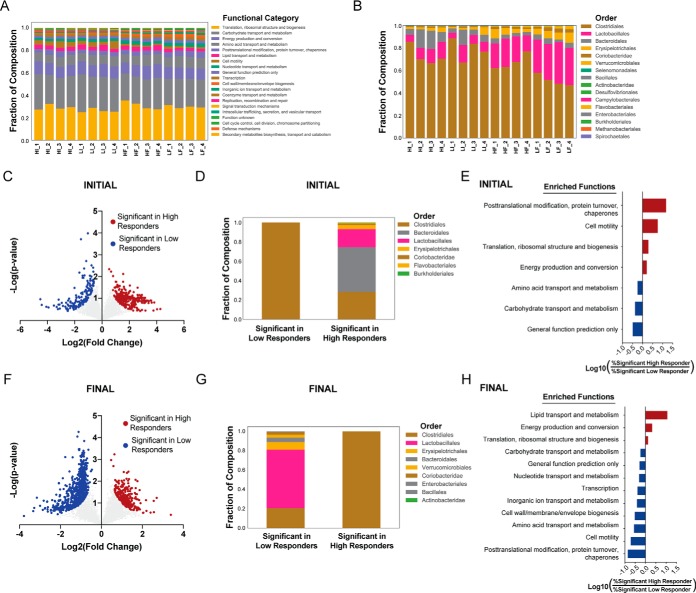
**Functional and taxonomic characterization of fecal metaproteome in low- and high-responder mice fed the obesogenic diet.** *A*, Functional Composition. *B*, Taxonomic Composition. *C–E*, Comparison of significant proteins from high and low responders at the initial time point. *C*, Volcano plot displaying the fold change and *t* test significance of each protein in the metaproteome. Significance was set at π-score > 1. *D*, Taxonomic composition of significant proteins. *E*, Barplots demonstrating the functional bias in significant proteins. Compositions of eggNOG annotations were compared between high and low responders and the log ratios of the high abundance categories (>10 proteins) are shown. *F–H*, Comparison of significant proteins from high and low responders at the final time point. Same analysis as (*C–E*) for the final time point. Sample names in (*A–B*) are annotated H for High Responder, L for Low Responder, then I for Initial time point, F for Final time point, with 1, 2, 3, or 4 for replicate number.

We next focused the analysis on discovering patterns in the fecal metaproteome that might have preceded or accompanied degree of responsiveness to the obesogenic diet. Toward this end, we examined the broad-scale functional composition of each sample's metaproteome through the eggNOG database. This revealed only modest variance among the samples (Fig. 4*A*). In contrast, viewing the composition of taxonomic orders in this manner revealed differences, both preceding and following diet exposure, that associated with a high- and low-response to the diet (Fig. 4*B*). There were 424 highly ranked proteins (π > 1) differentiating high and low responders at the initial time point (Fig. 4*C*). These proteins had large differences in their taxonomic origins with all proteins distinguishing the low responders belonging to Clostridiales whereas high responders had over 50% of proteins derived from Bacteroidales and Lactobacillales (Fig. 4*D*). Functionally, the proteins distinguishing high responders had a 14-fold enrichment in “Post-translational modification, protein turnover, and chaperones” proteins, and a 5.6-fold enrichment in “Cell motility” proteins (Fig. 4*E*). Many of the posttranslational modification, protein turnover, and chaperone proteins with the largest differences between high and low responders were chaperone proteins (supplemental Fig. S3*B*), a potential indication of a microbial stress response. The increased representation of cell motility proteins was a result of a subset of flagella, mostly derived from the order Clostridiales, that were significantly increased in high responders (*p* < 0.0001; supplemental Fig. S3*C*).

We also noted unique sets of carbohydrate metabolism and transport proteins differentially expressed at the initial time point. High responders had increased expression of Bacteroidale metabolism proteins including isomerases, kinases and aldolases, whereas Clostridiales uniquely had an increased expression of sugar transporters within low responders (supplemental Fig. S3*D*). This could be an indication of unique energy harvesting capacities in the microbiome present before the onset of HFD treatment (12).

In the samples collected following administration of the obesogenic diet, there were 970 proteins distinguishing high and low responders (Fig. 4*F*). In contrast to proteins discriminating responses at the onset of HFD exposure, the proteins corresponding to high response were derived entirely from Clostridiales, whereas nearly 60% of proteins in low responders were derived from Lactobacillales (Fig. 4*G*). Changes following the obesogenic diet associated with a high response to the diet included an 11-fold increased representation of lipid transport and metabolism proteins (Fig. 4*H*). It is possible that the increase in lipid metabolism proteins from Clostridiales mediates more efficient harvesting of energy from lipids in high responding mice.

##### Analysis of Fecal Mouse Proteins

**Fig. 5.**
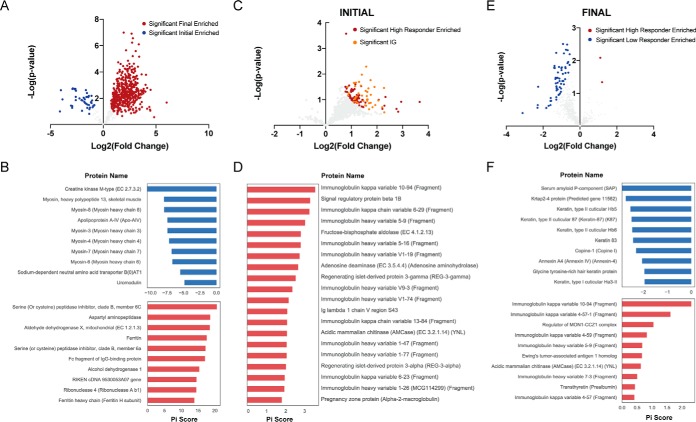
**Analysis of mouse fecal proteome.** After sub-setting the mouse derived proteins from the metaproteome data, differentially expressed proteins were determined using a statistical cut-off of π-score > 1. Volcano plots are shown demonstrating the log2 fold change (*x* axis) and log10 *p* value (*y* axis) for (*A*) differences between final and initial samples, (*C*) differences between high and low responders at the initial time point, and (*E*) differences between high and low responders at the final time point. The π-score of the most significant proteins from each analysis are shown below each volcano plot in bar plots (*B*, *D*, *F*).

In addition to analyzing microbial proteins from the metaproteome, we next subset the data to determine associations within the fecal mouse proteome. In total, 699 host proteins were quantified within all samples and therefore included in the statistical analyses. A large portion (77%) of mouse proteins were strongly influenced by HFD exposure, and 92% of those proteins were increased after HFD (Fig. 5*A*). Using DAVID functional enrichment (53), we identified significant (Bonferroni adjusted *p* values < 0.05) enrichment of digestion and myosin proteins before HFD treatment and mitochondrial proteins after HFD (supplemental Table S2). Notably, myosin proteins occupied 6 of the top 10 proteins associated with the initial samples (Fig. 5*B*). Phosphorylated myosin light chains have previously been linked to intestinal permeability after HFD exposure (5). Thus, our observed decrease in heavy chain myosin proteins may be related to changes in intestinal permeability. Regarding the increase of mitochondrial proteins, it was shown that HFD results in mitochondrial dysfunction (65), and our data likely reflects this phenomena.

We next looked for mouse fecal proteins that might discriminate high and low responders prior to HFD administration. Here, 109 mouse proteins strongly differed between the groups, all of which were enriched in the DIO prone mice (Fig. 5*C*). Of these proteins, 40 (37%) were related to immunoglobulin. This strong enrichment for immunoglobulin genes was confirmed through DAVID, which showed a significant, 3-fold enrichment (Bonferroni *p* value = 7.0E-11) for Immunoglobulin V-set proteins (supplemental Table S2). This enrichment of immunoglobulin variable domains was also illustrated in the top 20 proteins associated with a heightened response to HFD (Fig. 5*D*). These findings further illustrate the link between low-grade inflammation and DIO, as this is a potential indication of increased immune activity in high responder mice, before administration of HFD.

Applying the same analysis to samples collected after 8-weeks exposure to HFD also revealed interesting insight into proneness to HFD exposure. After the dietary intervention, 63 mouse proteins were highly ranked in their ability to discriminate between high and low responders. All but two of these proteins were enriched within the low responders (Fig. 5*E*). Functional analysis showed a significant 6-fold enrichment (Bonferroni *p* value = 4.0E-8) for keratin within the proteins enriched within low responders (supplemental Table S2). This increase of keratin could be an indication of greater colonic stress (66) in low responders at the final time point. High responders at this time had several immunoglobulin proteins within the top discriminatory proteins, though most were only modestly associated (Fig. 5*F*). Of note, many of the immunoglobulin proteins were among the strongest discriminatory proteins in high responders at both the initial and final day.

##### Analysis of Microbiota Composition Versus Proneness and Severity to DIO

**Fig. 6.**
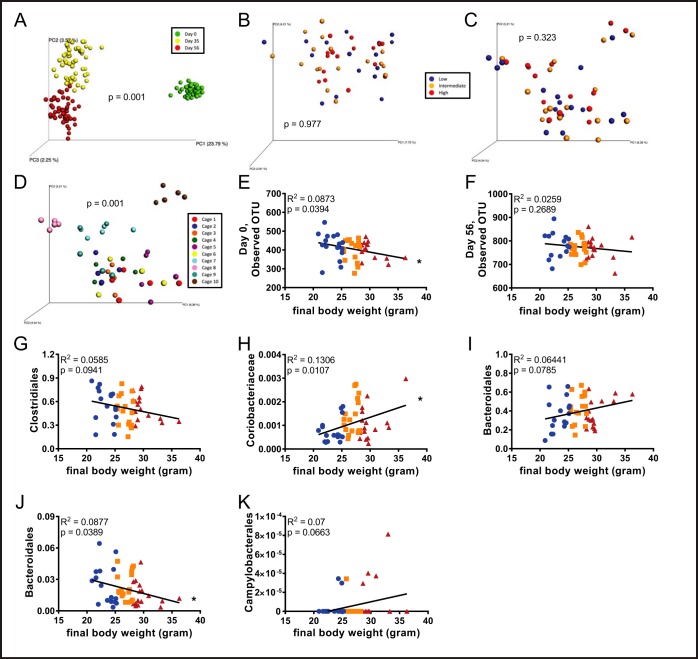
**Analysis of microbiota composition *versus* proneness and severity to DIO.** Fecal microbiota composition was analyzed using Illumina sequencing of the V4 region of 16S rRNA genes. Principal coordinates analysis (PCoA) was performed using the unweighted UniFrac distance metric (*A*) over the course of HFD administration, *B*, day 0, *C–D* day 56. Final body weights were correlated to alpha diversity at (*E*) day 0 and (*F*) day 56. Final body weights were correlated to bacterial groups found at the start of HFD administration: (*G*) Clostridiales, (*H*) Coriobacteriaceae, and (*I*) Bacteroidales. Final body weights were also correlated to bacterial groups found at the end of HFD administration: (*J*) Bacteroidales and (*K*) Campylobacterales. (*n* = 50). In *A–D*, categories were compared and statistical significance of clustering were determined *via* Permanova. In *E–K*, significance was determined using linear regression analysis (*p ≤ 0.005).

Last, we examined the potential of fecal microbiota composition, as analyzed by 16S rRNA gene sequencing to identify and/or reflect proneness to DIO. Visualization of fecal microbiota composition of all 50 mice at all time points by unweighted UniFrac revealed the expected dramatic difference in microbiota composition before and following administration of the obesogenic diet (*p* = 0.001; Fig. 6*A*). This analysis also showed clear, but much more modest differences between the 5 and 8-week post-dietary change time points (Fig. 6*A*). In contrast, using this approach to examine differences in beta diversity did not identify differences in microbiota composition in high or low-responders either prior to (*p* = 0.977; Fig. 6*B*), or following administration of the obesogenic diet (*p* = 0.323; Fig. 6*C*). Rather, in accord with other diets, 8-week administration of the diet, which provided mice an additional 8 weeks to share their microbiota with their cage-mates, we observed strong cage clustering of microbiota compositions (*p* = 0.001; Fig. 6*D*). Nonetheless, levels of alpha-diversity, prior to administration of the obesogenic diet were moderately but significantly correlated (r^2^ = 0.0873, *p* = 0.0394) with final body weights (Fig. 6*E*) suggesting that microbial community structure had some ability to predict proneness to DIO. An analogous but not significant trend was observed 8-weeks post-dietary change (Fig. 6*F*).

That overall assessment of microbiota composition lacked ability to identify high- and low- responder mice does not preclude the possibility that select OTUs might provide such power. Hence, we selected specific OTUs whose abundance was enriched or depleted at time 0 in the mice that developed the greatest degree of obesity in response to HFD. This yielded an array of bacterial groups, those of which had the ten lowest *p* values represented here, supplemental Fig. S4. However, determining whether these differences are reproducible and/or biologically significant will require further experimentation. Additionally, we examined the ability of bacterial candidates generated by the metaproteomic analysis to predict (day 0), or reflect (day 56), proneness to the obesogenic diet. Of the 12 taxa analyzed in the day 0, 3 groups showed correlations predicted by the proteomic analysis with *p* values lower than 0.1 (Fig. 6*G*–6*I*) whereas 9 did not (supplemental Fig. S5*A*–S5*I*). Regarding the taxa proteomic analysis identified as correlating with extent of obesity in the day 56 samples, 2 taxa correlated as determined with *p* values lower than 0.1 (Fig. 6*J*–6*K*) whereas the 10 others analyzed did not meet this criteria (supplemental Fig. S6*A*–S6*J*). Thus, overall, although development of approaches to predict proneness to obesity via analysis of the fecal proteome and/or microbiome remains a work in progress, these findings support its potential to contribute to such prognostications.

## DISCUSSION

The goal of this study was to improve understanding of non-genetic determinants of DIO, focusing on parameters that might be impacted by gut microbiota, which is known to play a role in dictating severity of DIO. As obesity is promoted by low-grade, systemic inflammation, which can be driven by exposure to microbiota products (5), we hypothesized that inflammatory and microbial factors might impact behavior and/or metabolism and thereby predict the extent of DIO displayed by individual hosts. However, the behavioral measures of general activity and anxiety in 6–8 week old mice were not predictive of susceptibility to HFD-induced obesity whereas the inflammatory markers IL-6, MPO, CXCL1, and LCN-2 showed only very limited ability in discriminating proneness to DIO when measured before, during, or after administration of HFD. From a microbial perspective, research has shown roles for LPS and flagellin in inflammation and obesity (14, 67, 68). However, although measuring flagellin levels showed promise for predicting weight after administration of HFD, it was less successful prior to administration. Our results revealed new evidence of host-microbial interactions underlying differential weight gain.

To find microbial factors that may correlate to DIO, we next turned to an untargeted metaproteomic approach. Our results confirm prior research showing large shifts in the overall structure of the metaproteome after administering HFD (50). These broad shifts seem to be driven by proteins derived from Clostridiales and Bacteroidales, which decreased upon exposure to HFD, whereas the composition of Lactobacillales proteins expands. Interestingly, this difference was not observable in our analysis of the microbiota by 16S sequencing. One possible explanation is the known discrepency between genomic and proteomic technologies (52) which is supported by the notion that differences in protein abundance are not directly associated with species composition because of complex regulatory processes. However, other DNA sequence-based studies have also shown significant alterations in Clostridiales and Bacteroidales upon exposure to HFD (5, 69), further suggesting a role for these taxonomies in HFD response.

Functionally, the most striking shift with HFD was the decreased abundance of flagella after administration of HFD. Flagellin proteins can be targeted in several ways by the host, including the release of anti-flagellin IgA and anti-flagellin IgG. The levels of Anti-flagellin IgA are anti-correlated with total flagellin load and are a key mechanism for down regulating motility-related genes (14). Although the overall levels of anti-flagellin IgG and IgA in general did not significantly correlate with the obesogenic outcomes, we did see a distinct immune signature within the fecal proteome of the high responder mice. This data may suggest that the mice that gain the most weight have a baseline immune reaction occurring before treatment. Possible antigens of this immune reaction were also identified from the metaproteome data including a subset of flagellin proteins that effectively discriminate high and low responders. However, as most of the identified immunoglobulin subunit regions could be a result of either IgA or IgV (70), it is not clear whether this potential reaction is mediated through TLR5, NOD-like receptor 4, or other mechanisms (67).

Taxonomic differences were also consistent with the idea that flagellin directed immunoglobulin may be shaping the gut of high responders before the onset of HFD. The majority of flagellin proteins identified were derived from Clostridiales, and Bacteroidales do not contain flagella (71). Here we observed a dominance of Clostridiales proteins enriched in low responders at the onset whereas Bacteroidales proteins contained a large portion of the high responder metaproteome. If the observed immunoglobulin proteins from the metaproteome were targeting flagella, it may be expected that the portion of Clostridiales proteins would be shifted in favor of Bacteroidales.

In total, our results suggest the ability of host and microbial proteomics to discern subjects particularly prone to developing DIO. Our results indicated significant metaproteome differences between high and low responding mice despite the limited number of samples analyzed. In addition, the taxonomic origins and functional roles of these discriminatory proteins suggested new evidence that host-microbiota interactions may be underlying proneness to DIO. Although larger studies are needed to confirm our results, the fecal metaproteome appears to be a promising tool for identifying hosts at risk of weight gain upon exposure to an obesogenic diet.

## Data Availability

All data generated or analyzed during this study are included in this published article. Metaproteomic data is available through massive (https://massive.ucsd.edu/) under study ID MSV000083891. The data is also available through Proteome Xchange (http://proteomecentral.proteomexchange.org) under the study ID PXD014128. Unprocessed sequencing data are deposited in the European Nucleotide Archive (https://www.ebi.ac.uk/ena) under accession number PRJEB33328.

## Supplementary Material

Table S1

Table S2

Supplemental Information

## Footnotes

^1^ The abbreviations used are:

DIO: diet-induced obesity
CXCL1: chemokine (C-X-C motif) ligand 1
ELISA: Enzyme-linked immunosorbent assay
FliC: flagellin
HEK: human embryotic kidney
IgA: Immunoglobulin A
IgG: Immunoglobulin G
IL-6: interleukin 6
Lcn-2: lipocalin-2
LPS: lipopolysaccharide
MPO: myeloperoxidase
MRI: magnetic resonance imaging
TLR4: Toll-like receptor 4
TLR5: Toll like receptor 5
TNF-α: tumor necrosis factor alpha
MS: mass spectrometry
TMT: Tandem Mass Tags.
