# Supplementary material for: Associations of the Fecal Microbial Proteome Composition and Proneness to Diet-induced Obesity
Source: Mol Cell Proteomics. 2019 Jul 1;18(9):1864–79. doi: 10.1074/mcp.RA119.001623 (PMC6731084; doi:10.1074/mcp.RA119.001623)
Supplement: Supplemental Information [file 153454_0_supp_345885_pswm2b.pdf]

SUPPLEMENTAL TABLES

Table S1. Protein relative abundance table. Normalized summed signal-to-noise ratios per sample are displayed for each protein identified. Also included is an indication of which database the protein was identified from, the taxonomy of the protein identification, the percent coverage of peptides associated with the protein and the number of unique peptides identified per protein.

Table S2. Functional enrichments within proteins associated with high fat diet responses. Annotated clusters of functional enrichments from the DAVID functional annotation clustering application are shown. Tabs separate the results from different mouse protein sets.

SUPPLEMENTAL FIGURE LEGENDS

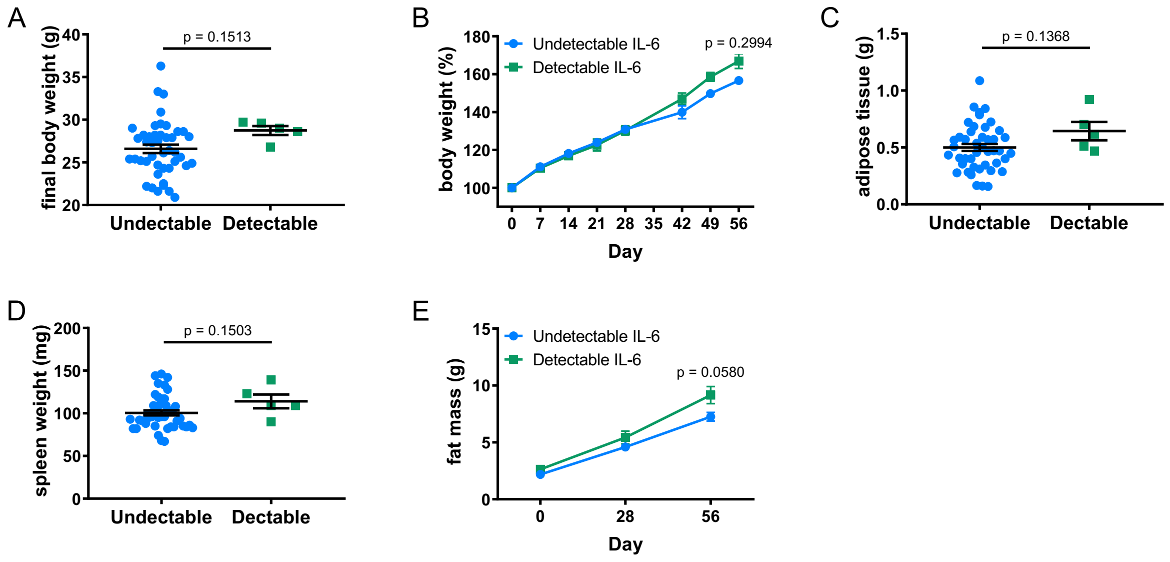

Supplemental Figure 1. Comparison of metabolic parameters in mice with detectable vs. undetectable basal serum IL-6. CXCL1 at day -7 were quantified using ELISA kits. Mice with detectable or undetectable levels of CXCL1 were compared by (**A**) final body weight, (**B**) body weight growth by

percent, (**C**) perigonadal adipose tissue, (**D**) spleen weight, and (**E**) fat mass measured by MRI. Data are the means +/- S.E.M. (*N*=45, 5 for Untectable IL-6 and Detectable IL-6 groups, respectively). Significance was determined using *t*-test. In **B** + **E**, statistical significance was determined by *t*-test at day 56.

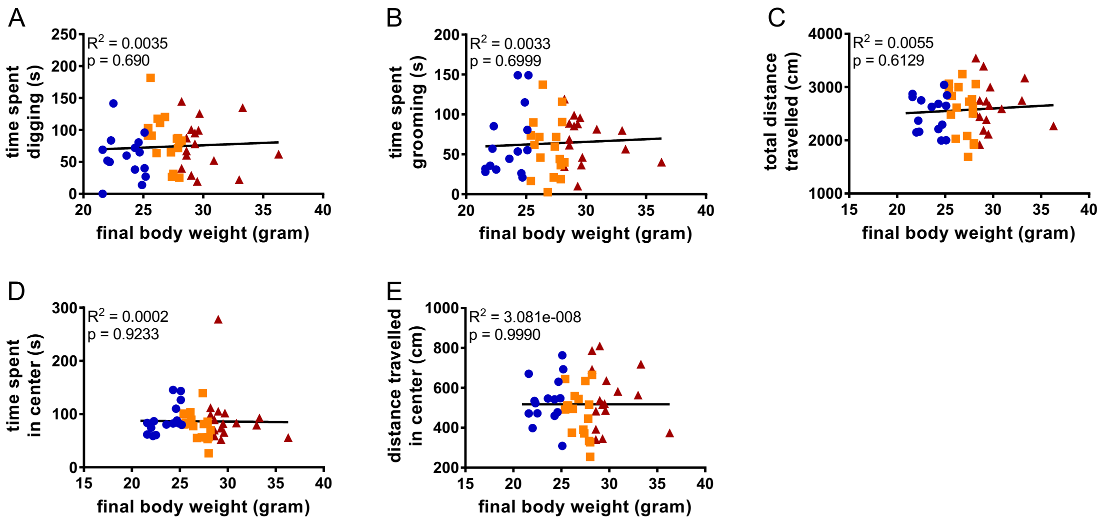

Supplemental Figure 2. Lack of association of behavioral measurements with proneness to DIO. Final body weights were correlated to home cage behaviors represented by (**A**) time spent digging, (**B**) time spent grooming, and (**C**) total distance travelled. Final body weights were also correlated to open field behaviors represented by (**D**) time spent in the center and (**E**) distance travelled in the center. (*N*=50). Significance was determined using linear regression analysis.

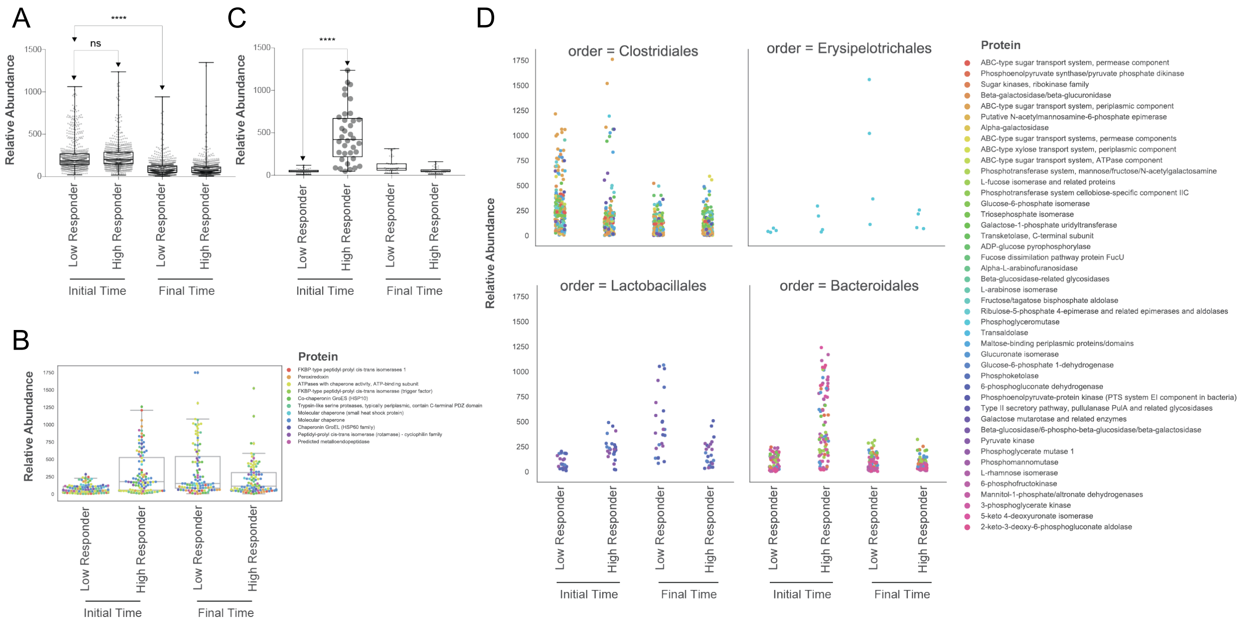

Supplemental Figure 3. Subsets of the metaproteome differentially expressed in DIO. (**A**) A combined boxplot and swarmplot displaying the relative abundances of all flagella proteins. (**B**) A combined swarm plot and boxplot showing the relative abundance of a subset of posttranslational modification and chaperone proteins significantly enriched (π-score > 1) in high responders at the initial time point. (**C**) A combined boxplot and swarmplot displaying the relative abundances of a subset of flagella proteins significantly enriched in high responder mice at the initial timepoint (π-score > 1). (**D**) Swarm plots subset by taxonomic order showing the relative abundance of carbohydrate transport and metabolism proteins differentially expressed (|π-score| > 1) between high and low responders at the initial time point. In **A** and **C**: ns, not significant; ****, ANOVA p-value < 0.0001.

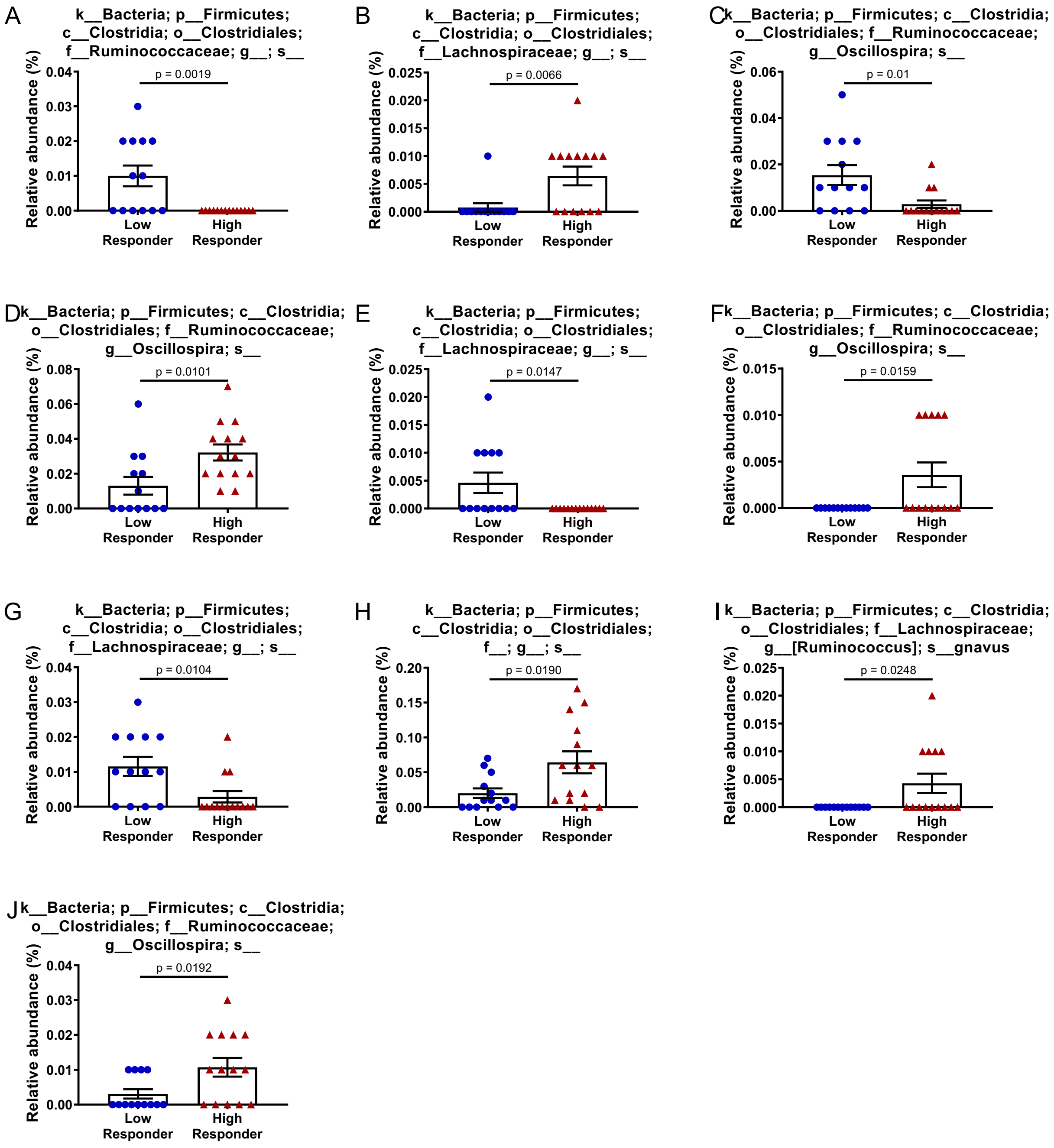

Supplemental Figure 4. Display of OTUs enriched or depleted in DIO prone and resistant mice. Fecal microbiota composition was analyzed using Illumina sequencing of the V4 region of 16S rRNA genes. 10 OTU with lowest uncorrected p value in t-test between low and high responders represented here. Data are the means +/- S.E.M. (*N*=50). Significance was determined using *t-test.*

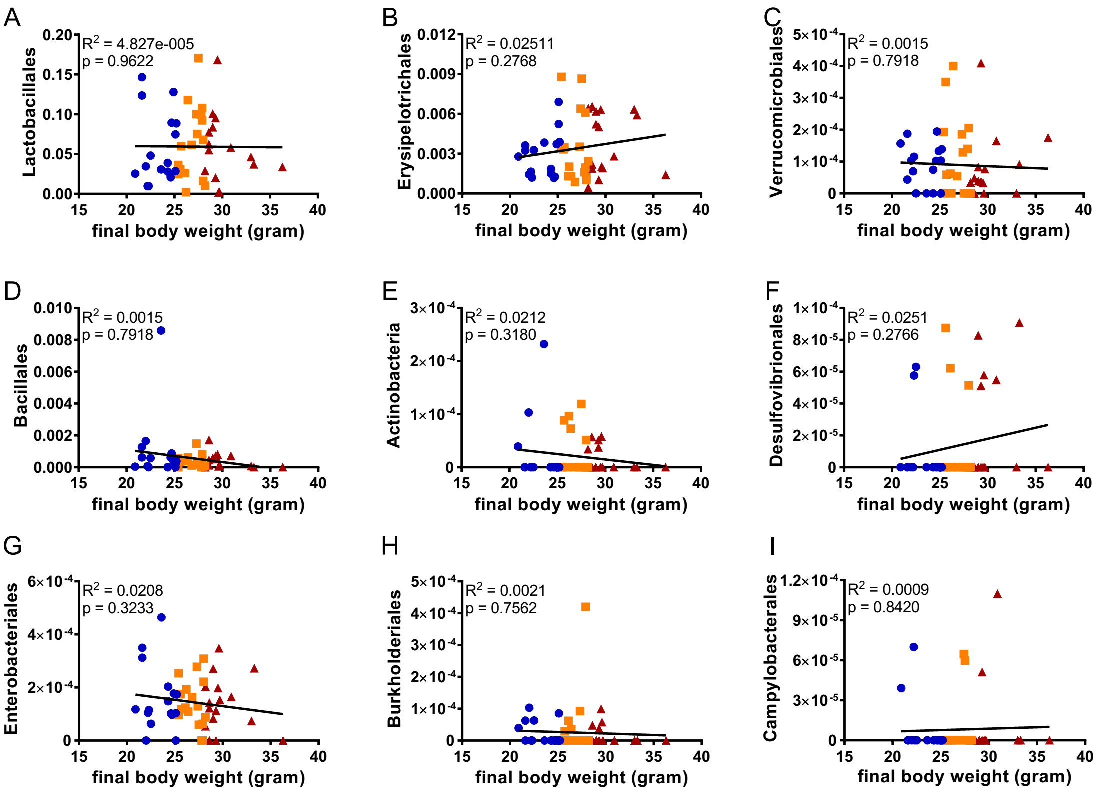

Supplemental Figure 5. Analysis of microbiota composition vs. development of DIO. Fecal microbiota composition was analyzed using Illumina sequencing of the V4 region of 16S rRNA genes. Final body weights were correlated to bacterial groups found at the start of HFD administration: (**A**) Lactobacillales, (**B**) Erysipelotrichale, (**C**) Verrucomicrobiales, (**D**) Bacillales, (**E**) Actinobacteria, (**F**) Desulfovibrionales, (**G**) Enterobacteriales, (**H**) Burkholderiales, and (**I**) Campylobacterales. (*N*=50). Significance was determined using linear regression analysis.

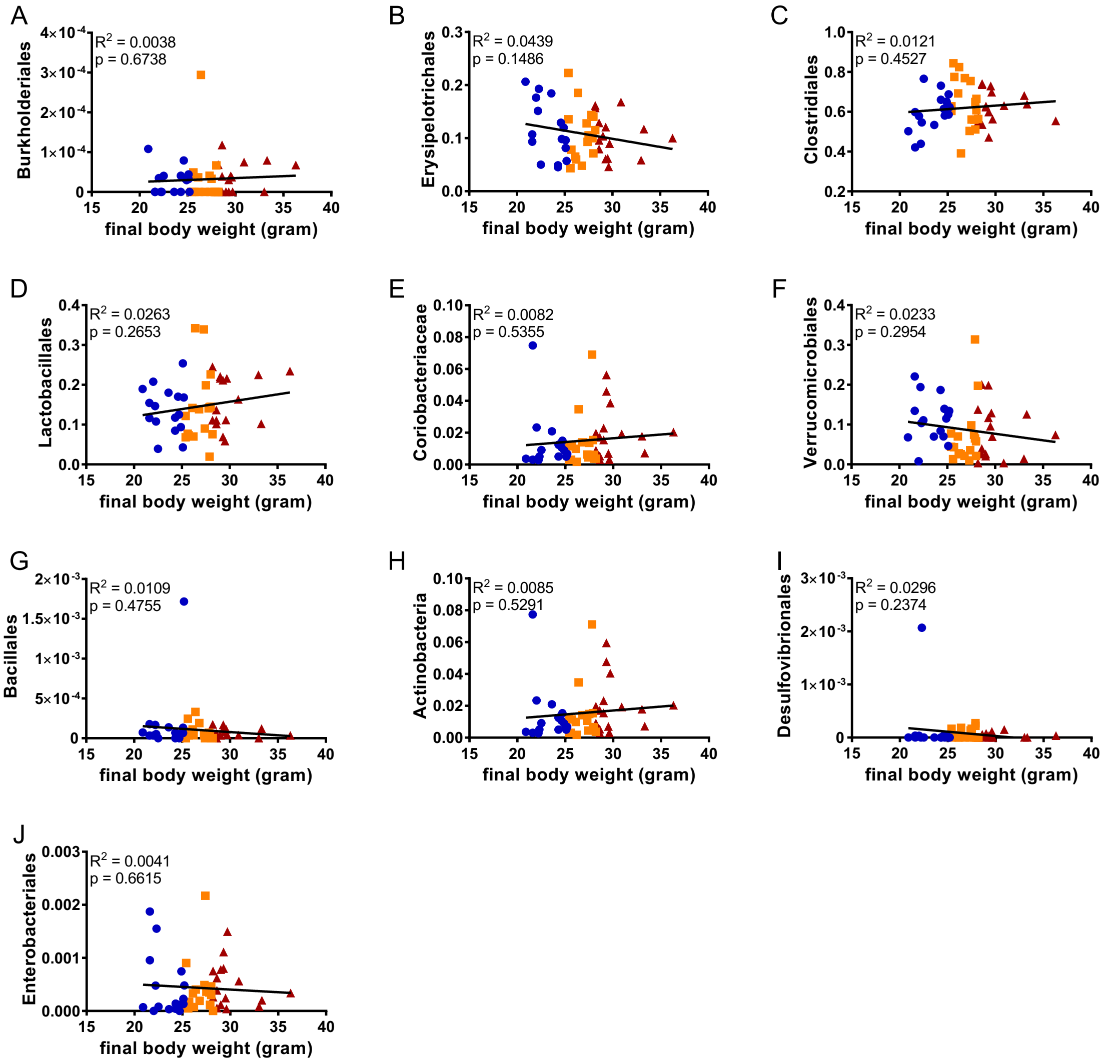

Supplemental Figure 6. Association of microbiota composition vs. severity of DIO. Fecal microbiota composition was analyzed using Illumina sequencing of the V4 region of 16S rRNA genes. Final body weights were correlated to bacterial groups found at the end of HFD administration: (**A**) Burkholderiales, (**B**) Erysipelotrichale, (**C**) Clostridiales, (**D**) Lactobacillales, (**E**) Coriobacteriaceae, (**F**) Verrucomicrobiales, (**G**) Bacillales, (**H**) Actinobacteria, (**I**) Desulfovibrionales, and (**J**) Enterobacteriales. (*N*=50). Significance was determined using linear regression analysis.
